# Preferential down-regulation of phospholipase C- **β** in Ewing's sarcoma cells transfected with antisense EWS-Fli-1

**DOI:** 10.1054/bjoc.1998.0870

**Published:** 1999-12-08

**Authors:** T Dohjima, T Ohno, Y Banno, Y Nozawa, Y Wen-yi, K Shimizu

**Affiliations:** Departments of Orthopaedic Surgery Biochemistry Gifu University School of Medicine, Tsukasamachi-40, Gifu, 500-8705, Japan

**Keywords:** phospholipase C, signal transduction, Ewing's sarcoma, antisense EWS-Fli-1, growth inhibition

## Abstract

EWS-Fli-1, a fusion gene found in Ewing's sarcoma and primitive neuro-ectodermal tumour (PNET), encodes a transcriptional activator and promotes cellular transformation. We have made stable Ewing's sarcoma cells expressing antisense EWS-Fli-1 transcripts by transfecting the antisense EWS-Fli-1 expression plasmid. These cells showed partial loss of endogenous EWS-Fli-1 proteins and suppression of the cell growth. To elucidate the molecular mechanisms underlying the growth inhibition, we examined the changes of signal transducing proteins by immunoblot analysis in Ewing's sarcoma cells stably expressing antisense EWS-Fli-1 transcripts. Western blotting of the cell proteins revealed that expressions of phospholipase Cβ2 and β3 (PLCβ2, PLCβ3), and also protein kinase C α and β (PKCα, β) were significantly reduced by transfecting with antisense EWS-Fli-1. The inositol phosphates production by bradykinin (BK), but not platelet-derived growth factor (PDGF), was suppressed in these cells. These results suggest that the PLCβ2 and PLCβ3 may play a role in tumour proliferation in Ewing's sarcoma cells. © 2000 Cancer Research Campaign


*British Journal of Cancer* (2000) **82**(1), 16–19
© 2000 Cancer Research Campaign
Article no. bjoc.1999.0870

### Preferential down-regulation of phospholipase C-β in Ewing's sarcoma cells transfected with antisense EWS-Fli-1

**T Dohjima[1], T Ohno[1], Y Banno[2], Y Nozawa[2], Y Wen-yi[1] and K Shimizu[1]**

Departments of [1]Orthopaedic Surgery and [2]Biochemistry, Gifu University School of Medicine, Tsukasamachi-40, Gifu 500-8705, Japan

**Summary** EWS-Fli-1, a fusion gene found in Ewing's sarcoma and primitive neuro-ectodermal tumour (PNET), encodes a transcriptional activator and promotes cellular transformation. We have made stable Ewing's sarcoma cells expressing antisense EWS-Fli-1 transcripts by transfecting the antisense EWS-Fli-1 expression plasmid. These cells showed partial loss of endogenous EWS-Fli-1 proteins and suppression of the cell growth. To elucidate the molecular mechanisms underlying the growth inhibition, we examined the changes of signal transducing proteins by immunoblot analysis in Ewing's sarcoma cells stably expressing antisense EWS-Fli-1 transcripts. Western blotting of the cell proteins revealed that expressions of phospholipase Cβ2 and β3 (PLCβ2, PLCβ3), and also protein kinase C α and β (PKCα, β) were significantly reduced by transfecting with antisense EWS-Fli-1. The inositol phosphates production by bradykinin (BK), but not platelet-derived growth factor (PDGF), was suppressed in these cells. These results suggest that the PLCβ2 and PLCβ3 may play a role in tumour proliferation in Ewing's sarcoma cells. © 2000 Cancer Research Campaign

**Keywords**: phospholipase C; signal transduction; Ewing's sarcoma; antisense EWS-Fli-1; growth inhibition

Karyotype analysis of Ewing family of tumours, which includes Ewing's sarcoma, primitive neuroectodermal tumours and Askin tumours, revealed characteristic translocations t(11;22) or t(21;22) (Whang-Peng et al, 1984; Sorensen et al, 1994), and such chromosomal rearrangement resulted in the expression of the aberrant fusion products which may be responsible for malignancy (Delattre et al, 1992; Rabbitts et al, 1994). Molecular analysis of these translocations revealed that 5′-region of EWS (from band 22q12) is fused to the 3′-region of Fli-1 gene (from band 11q24) or erg gene (from band 21q22). Functional characterization of the EWS-Fli-1 and EWS-erg chimaeric proteins suggested that they function as transcriptional activators (May et al, 1993; Ohno et al, 1993; Bailly et al, 1994). The extreme carboxyl terminal region of Fli-1 and erg protein is responsible for sequence specific DNA binding (Reddy et al, 1991; Rao et al, 1993). EWS protein was shown to be an RNA binding protein (Ohno et al, 1994), and the amino-terminal region of EWS protein was shown to function as a regulatory domain or as a transactivator domain depending on the target sequences used (May et al, 1993; Ohno et al, 1993; Bailly et al, 1994). Recently, some of the target genes modulated by the EWS-Fli-1 protein have been identified (Braun et al, 1995; May et al, 1997). Ewing's sarcoma cells transfected with antisense EWS-fusion expression plasmids were severely impaired in growth, colony formation, and tumorigenicity in nude mice (Ouchida et al, 1995). Antisense EWS-Fli-1 oligodeoxynucleotides against the fusion RNA were also shown to reduce the growth of the tumour cells significantly both in vitro and in vivo (Tanaka et al, 1997). Antisense DNA has been considered to inhibit the expression of protein products of the transcripts through several mechanisms (Haeuptle et al, 1986; Munroe, 1988; Walder and Walder, 1988).

It has been known that signal-transducing phospholipases and lipid-derived messengers may be involved in cell proliferation, and differentiation (Rhee, 1994; Singer et al, 1997). Phosphoinositide-specific phospholipase C (PI-PLC) generates inositol 1,4,5-trisphosphate (IP3) and diacylglycerol (DG) from phosphatidylinositol-4, 5 bisphosphate (PIP2). IP3 stimulates the release of $Ca^{2+}$ from intracellular stores and DG activates protein kinase C (PKC) (Berridge and Irvine, 1984; Nishizuka, 1986). PI-PLC isozymes comprise a family of ten distinct enzymes at least (Rhee, 1994; Rhee and Bae, 1997; Singer et al, 1997). Two major subclasses of the mammalian enzymes, PLCβ and PLCγ, have been shown to be activated through G protein-linked receptors such as bradykinin (BK) and tyrosine kinase-linked receptors such as platelet-derived growth factor (PDGF) respectively.

In the present study, for the better understanding of the EWS-Fli-1 function in terms of cellular signal transduction mechanism, we have examined the phosphatidylinositol signalling in Ewing's sarcoma cells stably transfected with antisense EWS-Fli-1 expression plasmid.

### MATERIALS AND METHODS

### Cell culture and cell labelling

Ewing's sarcoma cell line (TC 135) kindly gifted from Dr Triche (Univ. Southern California, CA, USA) were transfected with pcDNA expression vector carrying antisense EWS-Fli-1 and with empty pcDNA vector for control and were selected for G-418-resistant permanent cell lines as described previously (Ouchida et al, 1995). For the measurement of inositol phosphate, cells were

*Received 28 May 1998*
*Revised 5 July 1999*
*Accepted 8 July 1999*

*Correspondence to:* T Ohno labelled with [$^3$H]inositol (1 mCi ml$^{-1}$) in inositol-free minimum Eagle's medium containing 0.3% bovine serum albumin (BSA) for 36 h.

### Western blot analysis

Ewing's sarcoma cells (pcDNA-vector with or without antisense EWS-Fli-1) were grown in 100-mm dishes to near confluency and washed twice in $Ca^{2+}$/$Mg^{2+}$-free phosphate-buffered saline (PBS). Cells were solubilized with ice-cold lysis buffer (1% Triton X-100, 0.1% sodium dodecyl sulphate (SDS), 0.5% sodium cholate, 10 mM EDTA, 10 mM EGTA, 50 mM sodium chloride (NaCl), 25 mM HEPES, 1 mM phenylmethylsulphonyl fluoride, and 10 mg ml$^{-1}$ leupeptin, pH 7.40). Insoluble materials were removed by centrifugation at 14 000 ***g*** for 20 min at 4°C. Proteins (100 µg) were separated by SDS polyacrylamide gel electrophoresis (SDS-PAGE) according to the method by Laemmli (1970). Electrophoretic blotting onto nitrocellulose membrane was carried out as the procedure of Towbin (1979). The nitrocellulose membrane was incubated with antibodies against Fli-1, PLCβ1, β2, β3, β4, γ1, γ2, δ1, δ2, PKCα, β1, β2, γ, δ, ε, ζ, Gqα (Santa Cruz Biotechnology, Inc., CA, USA) and Gi2α (Kyowa Hako, Kyoto, Japan) overnight. The quantitative determination of the proteins were performed by a densitometer (Atto, Densitograph series 1).

### Measurement of inositol phosphates

The [$^3$H]inositol-labelled cells were washed two times with modified Krebs-Ringer buffer consisting of 125 mM NaCl, 5 mM potassium chloride (KCl), 1.2 mM $KH_2PO_4$, 1.2 mM $MgSO_4$, 2 mM calcium chloride ($CaCl_2$), 6 mM glucose, 0.1% BSA, 25 mM Hepes (pH 7.4), and 20 mM LiCl. Experiments were conducted by the adding agonists to the labelled cells, and the reaction was terminated by the addition of 0.5 ml of 10% perchloric acid to each well. Inositol phosphates were separated using Dowex AG1-X8 anion exchange resin (200–400 mesh, formate form, Bio-Rad Laboratories) as described elsewhere (Banno et al, 1994). Protein was determined with the Bio-Rad protein assay kit using BSA as standard.

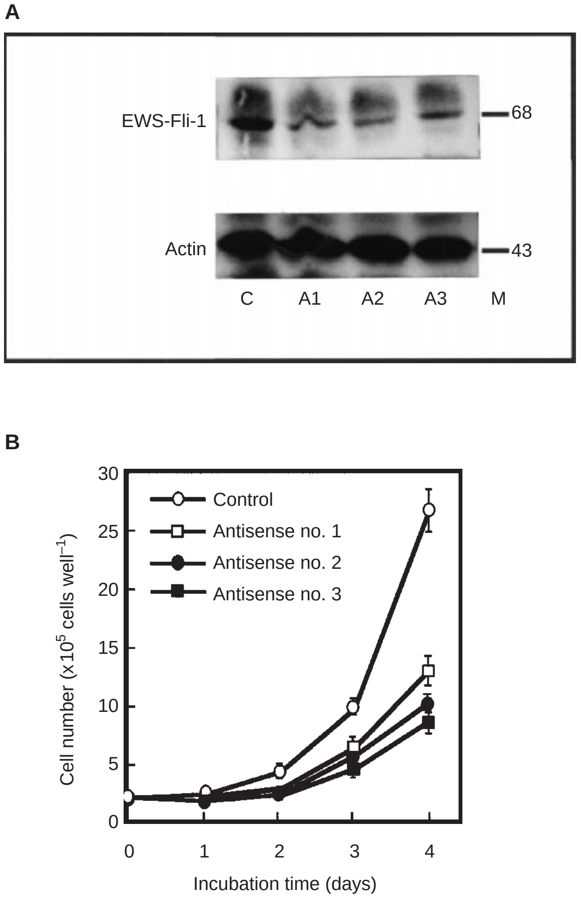

**Figure 1** Expression of EWS-Fli-1 protein and growth of Ewing's sarcoma cells (TC 135) transfected with empty vector (C, control) or antisense EWS-Fli-1 cDNA. (**A**) TC135 cells were lysed by sonication and the lysates (50 µg protein) were subjected to electrophoresis on 10% SDS-polyacrylamide gel, followed by immunostaining with antibody of Fli-1 protein. Lane 1, TC135 cells transfected with empty vector. Lanes 2, 3 and 4, TC135 cells transfected with anti EWS-Fli-1 cDNA (A1: cell line no. 1, A2: cell line no. 2, A3: cell line no. 3). (**B**) TC135 cells transfected with empty vector (control: ○) or antisense EWS-Fli-1 (cell lines no. 1: □, no. 2: ● and no. 3: ■) were plated at a concentration of $2 \times 10^5$ cells in 60 mm dishes, and incubated for the indicated times as described in Materials and methods. Cell numbers counted one day after plating were taken as zero time. Points represent as mean ± s.d. from duplicates determinations of two different experiments

### RESULTS AND DISCUSSION

We have transfected Ewing's sarcoma cell lines (TC135) with pcDNA expression vector carrying antisense EWS-Fli-1 cDNA and obtained ten G-418 resistant clones. These clones were characterized for the expression of antisense transcripts of EWS-Fli-1 by RT-PCR as we described before (Ouchida et al, 1995), with specific primers for the cytomegalovirus promotor in pcDNA and EWS, and selected three cell lines that highly expressed antisense EWS-Fli-1 transcripts confirmed by Northern blotting with RNA probe synthesized by in vitro transcription with EWS-Fli-1 expression plasmid (data not shown). In all of three cell lines, morphological changes were observed, e.g. elliptic shape and longer processes compared with the control small round cells. Western blotting analyses were carried out in these cell lines. These results revealed that there was a significant loss of expression of EWS-Fli-1 protein in Ewing's sarcoma cells transfected with antisense EWS-Fli-1 cDNA (Figure 1A). We have also compared the growth of these cell lines with TC 135 cells transfected with empty vector. The growth of Ewing's sarcoma cells expressing antisense EWS-Fli-1 was partially inhibited compared to the parent Ewing's sarcoma cells (Figure 1B).

Next we studied the production of inositol phosphates (IPs) in these cells. As shown in Figure 2, in Ewing's sarcoma cells transfected with or without antisense EWS-Fli-1, IPs production was stimulated by PDGF or BK. No significant difference in IPs production was observed in both cell types stimulated by PDGF. In contrast, IPs production induced by BK was considerably suppressed in the antisense EWS-Fli-1-transfected cells. These results suggest that the suppression of cell growth by transfecting antisense EWS-Fli-1 may be at least in part due to impairment of inositol lipid turnover mediated via G-protein coupled receptor activation rather than via tyrosine phosphorylation.

To examine further the mechanisms of reduced IPs production, immunoblotting was carried out in both cell types. Western blot analysis with antibodies for PLC isozymes, β (1, 2, 3, 4), γ (1, 2), δ (1, 2) revealed that PLCβ1, β2, β3, γ1, δ1, and δ2 were present in both cells (Figures 3 and 4), but that PLC-β4 and γ2 were not

*© 2000 Cancer Research Campaign*

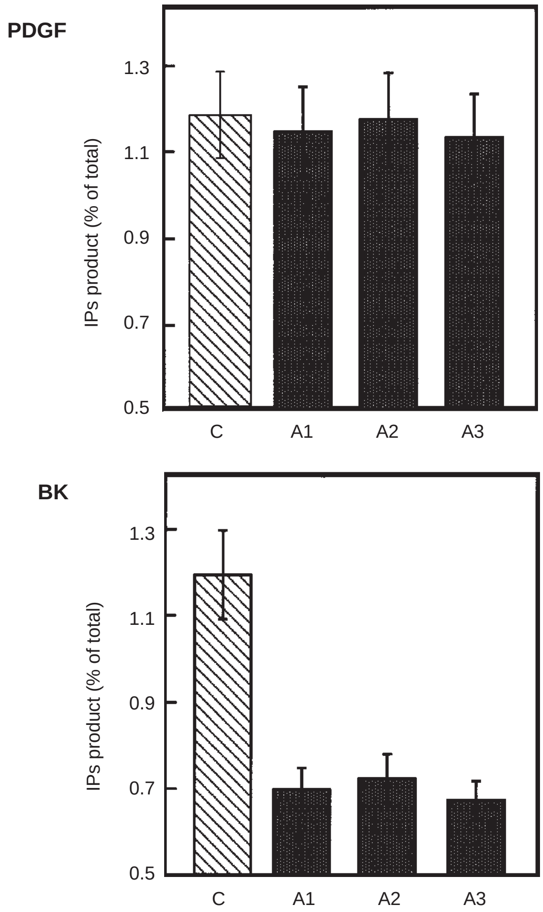

**Figure 2** Inositol phosphates production of Ewing's sarcoma cells (TC135) transfected with or without antisense EWS-Fli-1. TC135 cells transfected with pcDNA-vector (■: control) or with antisense EWS-Fli-1 (■: antisense, A1: cell line no. 1, A2: cell line no. 2, A3: cell line no. 3) were labelled with [$^3$H]myoinositol (1 μCi ml$^{-1}$) for 24 h. The labelled cells were stimulated with platelet-derived growth factor (PDGF, 10 ng ml$^{-1}$) for 10 min (**A**), or bradykinin (1 μM) for 1 min (**B**) in the presence of 25 mM LiCl. [$^3$H]Inositol phosphates were eluted through anion exchange column (AG1 × 8) by eluting with ammonium formate. The results were represented as means ± s.d. from triplicate determinations of two separate experiments

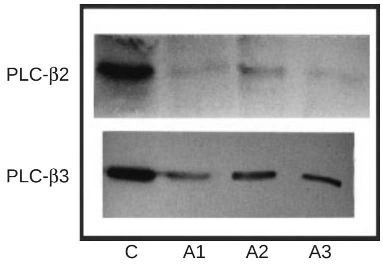

**Figure 3** Immunoblots of PLCβ2 and PLCβ3 expression in Ewing's sarcoma cells (TC135) transfected with or without antisense EWS-Fli-1. The cell lysates (100 μg proteins) were subjected to electrophoresis on 8% polyacrylamide gel and Western blot analysis was performed with anti-PLCβ2 and PLCβ3 antibodies. The molecular masses of the detected protein bands; PLCβ2, 140 kDa; PLCβ3, 155 kDa. Lane 1, TC135 cells transfected with empty vector. Lanes 2, 3 and 4, TC135 cells transfected with anti EWS-Fli-1 cDNA (A1: cell line no. 1, A2: cell line no. 2, A3: cell line no. 3). Data are representative of three experiments detectable (data not shown). No apparent differences were observed in PLC β1, γ1, δ1, and δ2 (Figure 4A). In contrast, to be

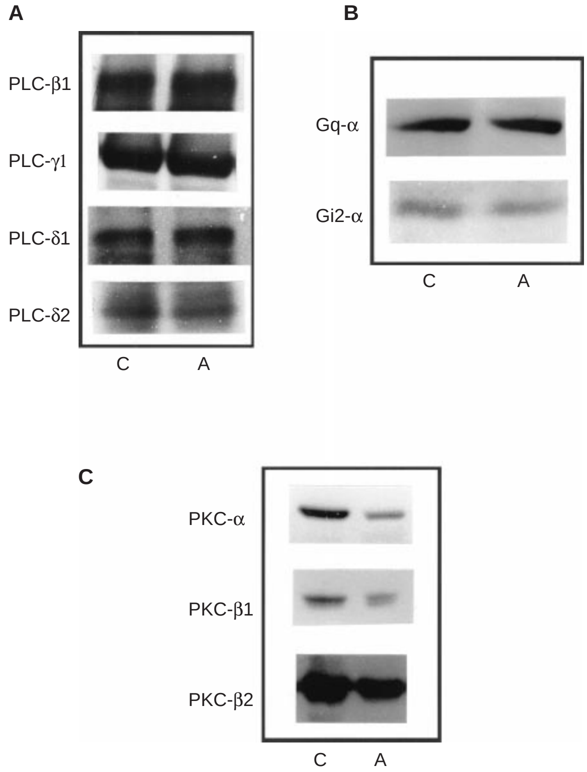

**Figure 4** Immunoblots of PLC isozymes, G proteins and PKC isozymes expression in Ewing's sarcoma cells transfected with or without antisense EWS-Fli-1. The cell lysates (100 μg proteins) were subjected to electrophoresis on 13% (for G proteins) and 8% (for PLC and PKC isozymes) polyacrylamide gels and western blot analysis was performed with antibodies against PLC and PKC isozymes and G proteins. The molecular masses of the detected protein bands; PLC-β1, 150 kDa; PLCγ1, 145 kDa; PLCδ1, 85 kDa; PLCδ2, 85 kDa. Gqα, 43 kDa; Gi2α, 42 kDa; PKCα, 81 kDa; PKCβ1, 82 kDa; PKCβ2, 80 kDa. (C: control cell, transfected with pcDNA-vector); (A: antisense cell, transfected antisense EWS-Fli-1/pcDNA). Data are representative of three experiments of great interest, PLC β2 and β3 were markedly down-regulated in the antisense-transfected cells (Figure 3) compared with the control cells. The quantitative determination of PLC isozymes showed that PLCβ2 and PLCβ3 in the antisense-transfected cells were reduced by 73% and 38% respectively.

PLC-βs are known to be activated by two G-protein families, one is Gi/o which is inhibited by pertussis toxins (PTX), and the other is Gq which is resistant to PTX. PLCβ1 and PLCβ3 are activated by a subunits of Gq, whereas PLC β2 is activated mainly by βα subunits of Gi/o (Rhee, 1994; Rhee and Bae, 1997; Singer et al, 1997). No significant differences in expression of Gqα and Gi2α were observed between both types of cells (Figure 4B). These results lead us to consider that suppression of inositol phosphates production in EWS-Fli-1 antisense-transfected cells may be due to the decrease of PLCβ2 and β3.

In addition, PKC isozymes were also examined, PKC isozymes were expressed in various levels in Ewing's sarcoma cells. Western blot analysis showed quantitative differences in PKCα, β1 and β2 between both cells (Figure 4C). The levels of PKCα, β1 and β2 were reduced by 44%, 57% and 30% in the transfected cells respectively, as compared with the control cells.

These results indicate that suppression of proliferation caused by transfecting the antisense EWS-Fli-1 could be at least in part due to impairment of the signalling pathway mediated by PLCβ2

and PLCβ3 activation and also by the subsequent activation of PKCα, β1, β2. Numerous studies have shown that PLCγ which is activated by tyrosine phosphorylation is involved in cell growth and carcinogenesis (Ji et al, 1997). On the other hand, it has been demonstrated that PLCγ activation is not essential for FGF receptor-mediating cell growth (Mohammadi, 1992; Peters et al, 1992). Furthermore, recent study has indicated essential role of PLCγ1 in mammalian growth (Ji et al, 1997). There have been some reports describing reduction or loss of expression of PLCβ isozymes in human diseases, e.g. loss of PLCβ3 gene expression in MEN1 disease (multiple endocrine neoplasia type 1) (Weber et al, 1994) and decreased PLCβ2 expression in abnormal platelet aggregation (Lee et al, 1996). Furthermore, recent study has demonstrated that expression of catalytically inactive PLCβ inhibits growth of small-cell lung cancer, indicating that signalling through Gq and PLCβ is a dominant pathway involved in the transformed growth of cancer cells (Beekman et al, 1998). In our study, we have shown that in Ewing's sarcoma cells expressing the antisense EWS-Fli-1 transcripts, the levels of PLCβ2 and PLCβ3 were much more decreased than the level of PLCγ1, suggesting that PLCβ2 and PLCβ3 may play an important role in cell proliferation in Ewing's sarcoma cells.

### ACKNOWLEDGEMENT

This study was supported by a Grant-in Aid from the Ministry of Science, Culture, Sports and Education of Japan.

### REFERENCES

Bailly RA, Bosselut R, Zucman J, Cormier F, Delattre O, Roussel M, Thomas G and Ghysdael J (1994) DNA-binding and transcriptional activation properties of the EWS-Fli-1 fusion protein resulting from the t(11;22) translocation in Ewing sarcoma. *Mol Cell Biol* **14**: 3230–3241

Banno Y, Okano Y and Nozawa Y (1994) Thrombin-mediated phosphoinositide hydrolysis in Chinese hamster ovary cells overexpressing phospholipase C-δ1. *J Biol Chem* **269**: 15846–15852

Beekman A, Helfrich B, Bunn PA and Heasley LE (1998) Expression of catalytically inactive phospholipase Cβ disrupts phospholipase Cβ and mitogen-activated protein kinase signaling and inhibits small cell lung cancer growth. *Cancer Res* **58**: 910–913

Berridge MJ and Irvine RF (1984) Inositol trisphosphate, a novel second messenger in cellular signal transduction. *Nature* **312**: 315–321

Braun BS, Frieden R, Lessnick SL, May WA and Denny CT (1995) Identification of target genes for the Ewing's sarcoma EWS/FLI fusion protein by representational difference analysis. *Mol Cell Biol* **15**: 4623–4630

Delattre O, Zucman J, Plougastel B, Desmaze C, Melot T, Peter M, Kovar H, Joubert I, De Jong P, Rouleau G, Aurias A and Thomas G (1992) Gene fusion with an ETS DNA-binding domain caused by chromosome translocation in human tumors. *Nature* **359**: 162–165

Haeuptle MT, Frank R and Dobberstein B (1986) Translation arrest by oligodeoxynucleotide complementary mRNA coding sequences yields polypeptides of predetermined length. *Nucleic Acid Res* **14**: 1427–1448

Ji QS, Winnier GE, Niswender KD, Horstman D, Wisdom R, Magnuson MA and Carpenter G (1997) Essential role of the tyrosine kinase substrate phospholipase C-γ1 in mammalian growth and development. *Proc Natl Acad Sci USA* **94**: 2999–3003

Laemmli UK (1970) Cleavage of structural proteins during the assembly of the head of bacteriphage T4. *Nature* **227**: 680–685

Lee SB, Rao AK, Lee KH, Yang X, Bae YS and Rhee SG (1996) Decreased expression of phospholipase C-β2 isozyme in human platelets with impaired function. *Blood* **88**: 1684–1691

May WA, Lessnick SL, Braun BS, Klemsz M, Lewis BC, Lunsford LB, Hromas R and Denny CT (1993) The Ewing's sarcoma EWS/FLI-1 fusion gene encodes a more potent transcriptional activator and is a more powerful transforming gene than FLI-1. *Mol Cell Biol* **13**: 7393–7398

May WA, Arvand A, Thompson AD, Braun BS, Wright M and Denny CT (1997) EWS/FLI-1-induced manic fringe renders NIH 3T3 cell tumorigenic. *Nature Genetics* **17**: 495–497

Mohammadi M, Dionne CA, Li W, Li N, Spivak T, Honegger AM, Jaye M and Schlessinger J (1992) Point mutation in FGF receptor eliminates phosphatidylinositol hydrolysis without affecting mitogenesis. *Nature* **358**: 681–684

Munroe SH (1988) Antisense RNA inhibits splicing of pre-mRNA *in vitro*. *EMBO J* **7**: 2523–2532

Nishizuyka Y (1986) Studies and perspectives of protein kinase C. *Science* **233**: 305–312

Ohno T, Rao VN and Reddy ESP (1993) EWS/Fli-1 chimeric protein is a transcriptional activator. *Cancer Res* **53**: 5859–5863

Ohno T, Ouchida M, Lee L, Gatalica Z, Rao VN and Reddy ESP (1994) The EWS gene, involved in Ewing family of tumors, malignant melanoma of soft parts and desmoplastic small round cell tumors, codes for an RNA binding protein with novel regulatory domains. *Oncogne* **9**: 3087–3097

Ouchida M, Ohno T, Fujimura Y, Rao VN and Reddy ESP (1995) Loss of tumorigenicity of Ewing's sarcoma cells expressing antisense RNA to EWS-fusion transcripts. *Oncogene* **11**: 1049–1054

Peters KG, Marie J, Wilson E, Ives HE, Escobedo J, Del Rosario M, Mirda D and Williams LT (1992) Point mutation of an FGF receptor abolishes phosphatidylinositol turnover and $Ca^{2+}$ flux but not mitogenesis. *Nature* **358**: 678–681

Rabbitts TH (1994) Chromosomal translocations in human cancer. *Nature* **372**: 143–149

Rao VN, Ohno T, Prasad DDK, Bhattacharya G and Reddy ESP (1993) Analysis of the DNA-binding and transcriptional activation functions of human Fli-1 protein. *Oncogene* **8**: 2167–2173

Reddy ESP and Rao VN (1991) erg, an ets related gene, codes for sequence-specific transcriptional activators. *Oncogens* **6**: 2285–2289

Rhee SG (1994) Regulation of phosphoinositide-specific phospholipase C by G protein. In *Signal-Activated Phospholipases*, Liscovitch M (ed) pp 1–30. RG Landes Company: Austin

Rhee SG and Bae YS (1997) Regulation of phosphoinositide-specific phospholipase C isozymes. *J Biol Chem* **272**: 15045–15048

Singer WD, Brown HA and Sternweis PC (1997) Regulation of eukaryotic phosphatidylinositol-specific phospholipase C and phospholipase *D. Annu Rev Biochem* **66**: 475–509

Sorensen PH, Lessmick SL, Lopez-Terrada D, Liu XF, Triche TJ and Denny CT (1994) A second Ewing's sarcoma translocation, t(21;22), fuses the EWS gene to another ETS-family transcription factor, ERG. *Nat Genet* **6**: 146–151

Tanaka K, Iwakuma T, Harimay K, Sato H and Iwamoto Y (1997) EWS-Fli1 antisense oligodeoxynucleotide inhibits proliferation of human Ewing's sarcoma and primitive neuroectodermal tumor cells. *J Clin Invest* **99**: 239–247

Towbin H, Staehelin T and Gordon J (1979) Electrophoretic transfer of proteins from polyacrylamide gels to nitrocellulose sheets: procedure and some applications. *Proc Natl Acad Sci USA* **76**: 4350–4354

Walder RY and Walder JA (1988) Role of RNase H in hybrid-arrested translation by antisense oligonucleotides. *Proc Natl Acad Sci USA* **85**: 5011–5015

Weber G, Friedman E, Grimmond S, Hayward NK, Phelan C, Skogseid B, Gogl A, Zedenius J, Sandelin K, Teh BT, Carson E, White I, Oberg K, Shepherd J, Nordenskjold M and Larsson C (1994) The phospholipase C β3 gene located in the MEN1 region shows loss of expression in endocrine tumours. *Hum Genet* **3**: 1775–1781

Whang-Peng J, Triche J, Knutsen T, Miset J, Douglass EC and Israel MA (1984). Chromosome translocation in peripheral neuroepithelioma. *N Engl J Med* **311**: 584–585

*© 2000 Cancer Research Campaign*
